# Genetic correlations between Alzheimer’s disease and gut microbiome genera

**DOI:** 10.1038/s41598-023-31730-5

**Published:** 2023-03-31

**Authors:** Davis Cammann, Yimei Lu, Melika J. Cummings, Mark L. Zhang, Joan Manuel Cue, Jenifer Do, Jeffrey Ebersole, Xiangning Chen, Edwin C. Oh, Jeffrey L. Cummings, Jingchun Chen

**Affiliations:** 1 Nevada Institute of Personalized Medicine, University of Nevada, Las Vegas 4505 S. Maryland Parkway, Las Vegas, NV 89154 USA; 2 Columbia University, West 116 St and Broadway, New York, NY 10027 USA; 3 Department of Biomedical Sciences, University of Nevada, Las Vegas, NV 89154 USA; 4 Center for Precision Health, School of Biomedical Informatics, The University of Texas Health Science Center at Houston, Houston, TX 77030 USA; 5 Laboratory of Neurogenetics and Precision Medicine, University of Nevada Las Vegas, Las Vegas, NV 89154 USA; 6 Department of Internal Medicine, UNLV School of Medicine, University of Nevada Las Vegas, Las Vegas, NV 89154 USA; 7 Department of Brain Health, School of Integrated Health Sciences, University of Nevada Las Vegas, Las Vegas, NV USA

**Keywords:** Genetics, Neuroscience

## Abstract

A growing body of evidence suggests that dysbiosis of the human gut microbiota is associated with neurodegenerative diseases like Alzheimer’s disease (AD) via neuroinflammatory processes across the microbiota-gut-brain axis. The gut microbiota affects brain health through the secretion of toxins and short-chain fatty acids, which modulates gut permeability and numerous immune functions. Observational studies indicate that AD patients have reduced microbiome diversity, which could contribute to the pathogenesis of the disease. Uncovering the genetic basis of microbial abundance and its effect on AD could suggest lifestyle changes that may reduce an individual’s risk for the disease. Using the largest genome-wide association study of gut microbiota genera from the MiBioGen consortium, we used polygenic risk score (PRS) analyses with the “best-fit” model implemented in PRSice-2 and determined the genetic correlation between 119 genera and AD in a discovery sample (ADc12 case/control: 1278/1293). To confirm the results from the discovery sample, we next repeated the PRS analysis in a replication sample (GenADA case/control: 799/778) and then performed a meta-analysis with the PRS results from both samples. Finally, we conducted a linear regression analysis to assess the correlation between the PRSs for the significant genera and the *APOE* genotypes. In the discovery sample, 20 gut microbiota genera were initially identified as genetically associated with AD case/control status. Of these 20, three genera (*Eubacterium fissicatena* as a protective factor*, Collinsella,* and *Veillonella* as a risk factor) were independently significant in the replication sample. Meta-analysis with discovery and replication samples confirmed that ten genera had a significant correlation with AD, four of which were significantly associated with the *APOE* rs429358 risk allele in a direction consistent with their protective/risk designation in AD association. Notably, the proinflammatory genus *Collinsella,* identified as a risk factor for AD, was positively correlated with the *APOE* rs429358 risk allele in both samples. Overall, the host genetic factors influencing the abundance of ten genera are significantly associated with AD, suggesting that these genera may serve as biomarkers and targets for AD treatment and intervention. Our results highlight that proinflammatory gut microbiota might promote AD development through interaction with *APOE*. Larger datasets and functional studies are required to understand their causal relationships.

## Introduction

Alzheimer’s disease (AD), the most common form of dementia, is a neurodegenerative disorder characterized by a multitude of pathological and clinical hallmarks such as a progressive decline in cognitive function and the buildup of toxic β-amyloid and tau proteins^1,2^. Due to the growing elderly population worldwide, the number of individuals with dementia is projected to reach 150 million globally by the year 2050^3^. Despite this growing burden on world health, the mechanisms underlying the disease pathology are not fully understood, impeding the development of optimally effective treatments^4^. Neuroinflammation has emerged as a key feature of AD with mechanistic and treatment implications due to the central role of microglia and inflammation in brain health^5,6^. There remains an urgent need to understand the genetic risk factors and pathological basis of neuroinflammation in AD so that individuals with a higher risk can be identified for earlier intervention.

Recently, an association between dysbiosis of the gut microbiome and neuroinflammation has been hypothesized to drive AD. The gut microbiota comprises a complex community of microorganism species that reside in our gastrointestinal ecosystem; alterations in the gut microbiota have been reported to influence not only various gut disorders but also brain disorders such as AD^7,8^. The human gut microbiota has been suggested to modulate brain function and behavior via the microbiota-gut-brain axis (MGBA), a bidirectional communication system connecting neural, immune, endocrine, and metabolic pathways^9^. Observational studies across multiple countries show reductions in gut microbiota diversity in AD patients compared to cognitively normal controls^10–12^. Current research indicates that bacteria populating the gut microbiota are capable of releasing lipopolysaccharide (LPS) and amyloids, which may induce microglial activation in the brain and contribute to the production of proinflammatory cytokines associated with the pathogenesis of AD^13^. The secretion of these biomolecules also harms the integrity of the MGBA and blood–brain barrier (BBB), which worsens with increasing dysbiosis^8,14^. The composition of the human gut microbiota and risk for AD have been suggested as heritable traits^2,15^. Apolipoprotein E ε4 (*APOE* ε4), the most well-established risk gene for AD, has recently been shown to correlate with microbiome composition in humans and mouse models of AD^16–18^. However, few studies have explored the correlation between *APOE* alleles and microbiome taxa at the human genomic level. In this study, we aim to determine the genetic correlation between the abundance of gut microbial genera and AD diagnosis. We further investigate whether gut microbial genera are correlated with *APOE* genotyping.

One promising approach to exploring this relationship is the use of polygenic risk score (PRS) analyses. A PRS is an overall estimate of an individual’s genetic liability for a specific trait. The software PRSice-2 is designed to calculate the PRS of an individual by aggregating and quantifying the effect of many single nucleotide polymorphisms (SNPs) in their genome, which are weighted by the effect sizes of each SNP derived from genome-wide association studies (GWASs)^19^. This approach has previously been used to explore the genetic relationship between gut microbial abundance and complex traits like bone mineral density, rheumatoid arthritis, and depression^20–22^. In the present study, we used this approach to determine the genetic relationship between 119 microbial genera and AD diagnosis. With the largest GWAS of the human gut microbiota^23^, we first conducted PRS analyses in an AD discovery sample to identify the genera genetically correlated with AD. We then verified our results in a replication sample and meta-analysis with the two samples. The correlation between the top ten significant genera and the *APOE* genotypes was further analyzed by linear regression analysis.

## Materials and methods

### Study design overview

**Figure 1:**
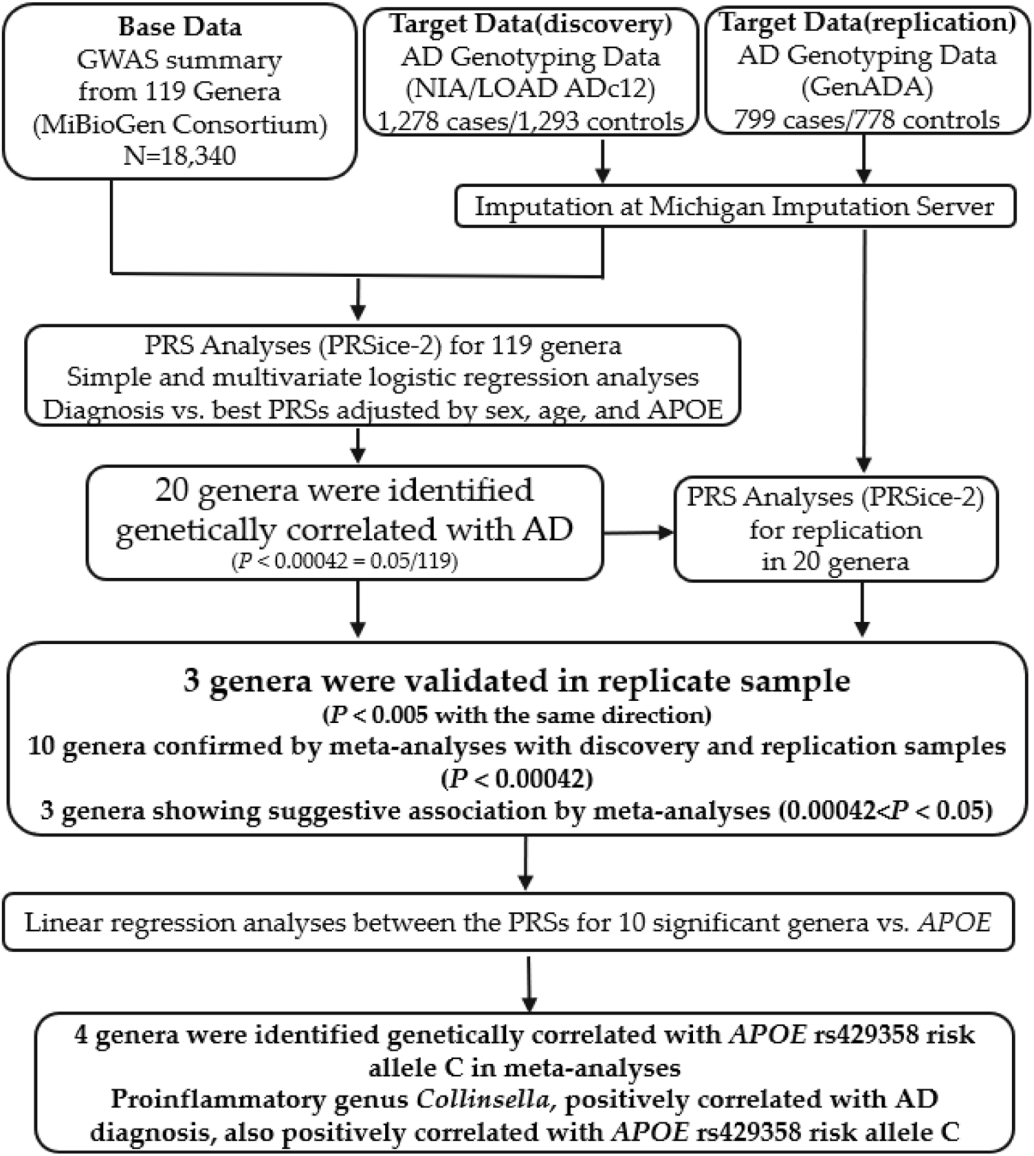
Study design flowchart. In the PRS analysis, “Base” data is used to provide effect sizes for SNPs shared with individuals in the “Target” data. Using PRSice-2, 20 genera were found to be significantly genetically associated with AD diagnosis in the discovery sample. Three genera were validated in the replication sample, and ten were confirmed by a meta-analysis from discovery and replicate samples. Linear regression analyses were used to determine the genetic correlation between the PRSs for ten significant genera and *APOE* genotyping. Three genera were identified as genetically correlated with *APOE* rs429358 risk allele C.

The overall design of our study is shown in Fig. 1. Briefly, we used PRSice-2^19^ to calculate PRSs for individuals from our discovery sample. PRSs were calculated based on the summary statistics for 119 microbial genera from the MiBioGen consortium. The significant association between genera and AD diagnosis was determined when the “best-fit” PRS model had a Bonferroni-corrected *p* < 0.00042 (0.05/119 = 0.00042). We then replicated the results in an independent sample. We conducted logistic regression analyses between the PRSs of associated genera and AD diagnosis to generate relative odds ratios (ORs) for meta-analysis. The multivariate logistic regression model was used to determine whether sex, age, and *APOE* genotypes affected the correlation between the PRSs of the associated genera and AD diagnosis. Furthermore, we conducted a linear regression analysis to evaluate the genetic association between the PRSs of ten significant genera and the *APOE* genotypes of individuals in our discovery and replication samples. This study was approved by our institutional review board (IRB) at the University of Nevada Las Vegas (UNLV).

### Data sources

#### Microbiome GWAS summary statistics (base data)

For our “base” GWAS data, we obtained summary statistics from the MiBioGen consortium initiative (www.mibiogen.org)^23^, which is the largest, multi-ethnic genome-wide meta-analysis of the gut microbiome to date (Table 1). The data includes 24 multi-ethnic cohorts comprising 18340 participants. 16S rRNA sequencing profiles from each individual were utilized to characterize their gut microbiota abundance using SILVA as a reference database^24^. The MiBioGen cohorts used a variety of platforms for genotyping their participants, such as the Illumina OmniExpress, Affymetrix 6.0, and more, which are detailed in the supplements of the original study^23^. The genotyping data from 23 cohorts were imputed at the Michigan Imputation Server (https://imputationserver.sph.umich.edu)^25^, while another genotyping data were imputed with IMPUTE2 software (v2.3.2)^23^. From the phylum to genus level, 31 loci were associated with gut microbiota taxa abundance (mbQTL, n = 20) or the presence/absence of taxa (mbBTL, n = 11) at the genome-wide significant threshold (*p* < 5.0 × 10^−8^)^23^. The SNP effect sizes reported in the mbQTL GWAS summary statistics represent how the host genetic loci affect the relative abundance of each microbiome taxa (mbQTLs)^23^. In the present study, we limited our analyses to the mbQTL summary statistics from the 119 microbial genera, as 16S rRNA sequencing correlates more accurately with the functional role of gut microbiota at lower taxonomic levels^26^.

**Table 1:** Information for studies used in our analyses.

Variable	Consortium or study	PMID	Year	Total sample size	Ethnic groups
Gut microbiota	MiBioGen	33462485	2021	18340	European (13266) Admixed (2571) Hispanic (1097) East Asian (811) Middle-East (481) African American (114)
Alzheimer’s disease	ADc12	19001172	2008	2571	European (2320) African American (251)
	dbGaP phs000168.v2.p2 NIA/LOAD consent 1 and 2			1278 cases/1293 controls	
	GenADA	17998437 19013250	2008	1577	European (1577)
	dbGaP phs000219.v1.p1			799 cases/778 controls	

Information for the data used in this study. The Multi-ethnic cohorts of the MiBioGen study include 16 European cohorts (n = 13266), one Middle Eastern cohort (n = 481), one East Asian cohort (n = 811), one American Hispanic/Latin cohort (n = 1097), one African-American cohort (n = 114), and four cohorts of multi-ancestry individuals (n = 2571). The Multi-ethnic cohort of the NIA/LOAD study contains African-American (n = 251) and Caucasian individuals (n = 2320).

#### AD genotyping data (target data): discovery and replication samples

For AD genotyping data we requested two datasets from dbGaP (https://www.ncbi.nlm.nih.gov/gap/), including the National Institute of Aging/Late-onset Alzheimer’s Disease Study (NIA/LOAD) cohort consents 1 and 2 (ADc12) (dbGaP phs000168.v2.p2)^27^, and the Multi-Site Collaborative Study for Genotype–Phenotype Associations in Alzheimer’s Disease Study (GenADA) (dbGaP phs000219.v1.p1)^28,29^. The ADc12 data were used as the discovery sample, and the GenADA data were used as the replication sample.

In this our study, AD cases were considered as any individual with dementia diagnosed with definite, probable, or possible AD at any point in their clinical course, according to the Criteria proposed in 1984 by the National Institute of Neurological and Communicative Disorders and Stroke, and the Alzheimer's Disease and Related Disorders Association (NINCDS-ADRDA)^30^. Included controls were neurologically evaluated individuals who were age-matched cognitively normal. Unspecified dementia, unconfirmed controls, and controls with other neurological diseases from the original studies were removed for our analyses, resulting in 1278/1293 cases/controls in the discovery sample ADc12, and 799/778 cases/controls in the replication sample GenADA. Demographic characteristics of the ADc12 and Gen/ADA samples are listed in Table 2, along with two major *APOE* SNP genotype information. More detailed descriptions of the data can be found in previous studies^27–29^.

**Table 2:** Demographic characteristics of the target data (ADc12 and GenADA) with *APOE* SNP genotyping.

			Discovery Sample (ADc12)			Replication Sample (GenADA)		
			Cases	Controls	Total	Cases	Controls	Total
Age, mean ± SD			1278	1293	2571	799	778	1577
			76.57 ± 6.71*	70.27 ± 10.29		72.24 ± 8.41**	73.40 ± 7.92	
Sex (Male/Female)			443/835	471/822	914/1657	339/460	276/502	615/962
*APOE* SNP Genotype	rs429358	T/T, n(%)	409 (32.0)	847 (65.5)	1256 (48.9)	296 (37.0)	589 (75.7)	885 (56.1)
		T/C, n(%)	682 (53.4)	414 (32.0)	1096 (42.6)	397 (49.7)	177 (22.8)	574 (36.4)
		C/C, n(%)	187 (14.6)	32 (2.5)	219 (8.5)	106 (13.3)	12 (1.5)	118 (7.5)
		C/C, n(%)	1206 (94.4)	1128 (87.2)	2334 (90.8)	739 (92.5)	661 (85.0)	1400 (88.8)
	rs7412	C/T, n(%)	71 (5.6)	159 (12.3)	230 (8.9)	60 (7.5)	113 (14.5)	173 (11.0)
		T/T, n(%)	1 (0.0)	6 (5.1)	7 (0.3)	0 (0.0)	4 (0.5)	4 (0.3)

Both discovery (ADc12) and replication (GenADA) samples were downloaded from dbGaP. Age, mean ± SD. For each case, the “Age” was the age at onset (AAO). For each control, the “Age” was the age at examination (AAE). *p = 5.97 × 10^−71^ when AAO compared to AAE in the discovery sample. **p = 4.94 × 10^−3^ when AAO compared to AAE in the replication sample.

The ADc12 genotyping data were originally generated with the Illumina Human610 QuadV1-B platform at 601273 SNPs, and the GenADA genotyping data with the Affymetrix 500k Set (Mapping 250k_NSP and Mapping 250k STY arrays).To maximize genetic variants, we conducted imputation for both discovery and replication samples at the Michigan Imputation Server (minimac4) (https://imputationserver.sph.umich.edu)^25^. The 1000 Genome Phase 3v5^31^ was used as a reference. After the imputation, standard quality control was performed with the Plink command (--maf 0.01 --hwe 1e-6 --geno 0.01 --mind 0.01)^32,33^. The final datasets were composed of 2571 individuals with 9997692 SNPs in the discovery sample, and 1577 individuals with 8914585 SNPs in the replication sample.

### Polygenic risk score (PRS) analyses via PRSice-2 software

PRSice-2 was mainly designed to calculate PRSs for individuals based on GWAS summary statistics data using the traditional “Clumping + Thresholding” (C + T) approach^19^. A key assumption of the C + T approach is that the SNPs comprising the PRS are independent of each other, which is controlled by thinning SNPs in linkage disequilibrium (LD) and retaining those that are the most significant (“Clumping”)^34^. SNPs are then thresholded by their *p*-values from the summary statistics, and the PRS is calculated for individuals at each threshold (“Thresholding”).

One major application of PRSice-2 is to evaluate the genetic correlation between different traits when provided GWAS summary statistics data from a base trait (base data) and genotyping data from a target trait (target data)^19^. The PRS itself is a numerical approximation of genetic liability for the base trait in the individuals in the target trait, based on their number of alleles from the target data and effect sizes drawn from the base data for a set of SNPs^35^. As mentioned above, the base (GWAS) data were from the 119 gut microbiome genera in the MiBioGen consortium study^23^. The target data were the discovery sample ADc12^27^ and the replication sample GenADA^28,29^. In this study, we first calculated PRSs for the 119 gut microbiome genera in the discovery sample ADc12 to determine which genera were genetically correlated with AD diagnosis. The best PRS model for each genus was calculated using the “best-fit” model implemented in the PRSice-2 program. For this purpose, a range of *p*-value thresholds applied to the base data, as well as the association *p*-value between the PRSs of each genus and AD diagnosis. For this purpose, a range of *p-*value thresholds was set from 5 × 10^−8^ to 1 with an incremental interval of 0.00005 (--interval 0.00005 --lower 5e-08) with LD clumping (--clump-kb 250 kb --clump-p 1.0 --clump-r2 0.1)^19^. In the discovery sample, a genus was considered significant if its assocation *p*-value from the “best-fit” model was less than 4.20 × 10^−4^ (0.05/119 with Bonferroni correction). To validate the significantly associated genera from the discovery sample ADc12, we conducted the same PRS analyses for them in the replication sample GenADA.

### Logistic regression and meta-analysis

To further evaluate the overall association of the 20 significantly associated genera from the discovery sample, we z-score normalized the "best-fit" PRSs from both the discovery sample ADc12 and replication sample GenADA. We then performed a simple logistic regression analysis for both samples between the normalized PRSs from the “best-fit” threshold for AD diagnosis using the glm function from the R package stats^36^.

Next, we conducted a random effects meta-analysis from both samples using the R package metafor v3.8-1^37^. The summary effect estimate of this meta-analysis identified ten significant genera that were used for all future analyses. Forest plots were generated to visualize the overall AD protective and risk effects across the significant genera using the “forestplot” R package^38^. To compare the normalized PRSs for the ten significant genera between AD cases and controls in the discovery sample, we conducted the unpaired Wilcoxon Rank Sum test with the wilcox.test function in R (v4.2.0)^36^ and visualized the results with box plots. Box plots were generated using the R program ggplot2 v.3.3.6^39^.

To account for potential confounding variables in our analysis, multivariate logistic regression was conducted between AD diagnosis and z-score normalized PRSs for significant microbial genera using the glm function from the R stats package^36^. Sex, age, and *APOE* genotypes (rs429358, rs7412) were used as covariates.

### Linear regression analyses between *APOE* genotypes and PRSs for the ten significant genera

Two *APOE* SNPs, rs429358 minor allele C and rs7412 major allele C, are well-known risk factors for AD^40,41^. We performed linear regression analyses to determine the genetic correlation between the two *APOE* SNPs and the normalized PRSs of the ten significant genera from the meta-analysis. The association was further evaluated by linear regression analysis adjusted for sex and age. All linear regression was performed using the lm function from the R stats package. Box plots with the ANOVA test (state compare means function) were created using the R packages ggplot2 (v3.3.6), ggpubr (v0.4.0), and stats (v0.1.0)^36,39^.

### Statistical analyses

The *p-*value threshold for significant association in the discovery sample and meta-analysis was set as *p* < 4.20 × 10^−4^ (0.05/119 with Bonferroni correction). For the replication sample, one-side significant level *p* < 0.005 (0.1/20 with Bonferroni correction) was used. For all other statistical analyses, such as linear regression analysis, the ANOVA test, and Wilcoxon Rank Sum test, *p* < 0.05 was considered significant. The Wilcoxon Rank Sum method, also known as the Mann–Whitney test, is a non-parametric alternative to the unpaired two-sample t-test, which can be used to compare two independent groups of samples without knowing their distribution^42^. The ANOVA method was utilized to test the association between the normalized PRSs for the ten significant genera and *APOE* genotypes^43^.

### Ethical approval and consent to participate

We are using the existing data for this study. Informed consent was obtained from all subjects and/or their legal guardian(s) in the original studies. Contributing studies received ethical approval from their respective institutional review boards (IRB). This study was performed per the Declaration of Helsinki and approved by the IRB at the University of Nevada Las Vegas (IRB #00002305, 10/12/2021).

## Results

### PRSs for ten microbiome genera were significantly associated with AD diagnosis

We first calculated the PRSs for the 119 microbiome genera for each individual from the discovery sample (ADc12) using the PRSice-2 program^19^. We found that 20 out of the 119 genera were significantly associated with AD diagnosis using the “best-fit” model (*p* < 4.20 × 10^−4^) (Table 3). Among these top 20 significant genera, six were identified as likely risk genera and 14 potentially protective genera for AD diagnosis. Risk genera included *Alistipes* and *Bacteroides* from the Bacteroidetes phylum, *Lachnospira* and *Veillonella* from the Firmicutes phylum, and *Collinsella* and *Sutterella* from the Actinobacteria and Pseudomonadota phyla, respectively. The most significant risk genus was *Bacteroides* (R^2^ = 0.011, *p* = 3.32 × 10^−6^) at the “best-fit” *p-*value threshold of 0.179 with 71984 SNPs. For protective genera, eleven out of fourteen were from the Firmicutes phylum (*Anaerostipes, Candidatus Soleaferrea, Catenibacterium, Eisenbergiella, Eubacterium coprostanoligenes group, Eubacterium fissicatena group, Eubacterium nodatum group, Intestinibacter, Lachnospiraceae UCG-008, Oscillibacter,* and *Roseburia),* two were from Actinobacteria (*Adlercreutzia* and *Gordonibacter*), and one was from Bacteroidetes (*Prevotella 9*). The most significant protective genus was *Intestinibacter* (R^2^ = 0.015, *p* = 1.01 × 10^−7^) at the “best-fit” *p-*value threshold of 0.190 with 70292 SNPs.

**Table 3:** Association between significant microbiome genera and AD diagnosis from “best-fit” PRSice-2 model.

Genera	Samples	Threshold	R^2^	*P*	Coeff	SE	SNP#	SampleSize
Adlercreutzia*	ADc12	0.3705	0.0068	**3.57E−04**	− 1870.66	524.00	101798	2571
	GenADA	0.0020	0.0036	3.91E−02	− 224.26	108.71	1684	1577
Alistipes	ADc12	0.1676	0.0080	**1.04E−04**	2581.83	665.28	67574	2571
	GenADA	0.3246	0.0023	1.03E−01	− 2953.44	1810.61	99785	1577
Anaerostipes	ADc12	0.0032	0.0078	**1.18E−04**	− 469.15	121.86	2834	2571
	GenADA	0.2989	0.0054	1.18E−02	4134.46	1641.01	96076	1577
Bacteroides*	ADc12	0.1792	0.0114	**3.32E−06**	3930.96	845.35	71984	2571
	GenADA	0.0009	0.0037	3.68E−02	221.55	106.10	922	1577
Candidatus Soleaferrea*	ADc12	0.0002	0.0090	**3.48E−05**	− 71.29	17.22	177	2571
	GenADA	0.0199	0.0020	1.28E−01	− 404.97	265.96	12431	1577
Catenibacterium	ADc12	0.3839	0.0082	**8.54E−05**	− 1276.68	324.95	71434	2571
	GenADA	0.0831	0.0080	2.11E−03	1068.34	347.56	25728	1577
**Collinsella**	ADc12	0.0002	0.0073	**1.78E−04**	125.63	33.51	230	2571
	GenADA	0.0003	0.0143	**4.36E−05**	229.15	56.06	335	1577
Eisenbergiella*	ADc12	0.1049	0.0082	**9.64E−05**	− 858.39	220.13	42727	2571
	GenADA	0.0439	0.0066	5.28E−03	− 1131.73	405.70	22002	1577
Eubacterium coprostanoligenes	ADc12	0.1020	0.0086	**7.19E−05**	− 1085.22	273.37	48130	2571
	GenADA	0.0101	0.0038	3.36E−02	707.65	333.01	7483	1577
**Eubacterium fissicatena**	ADc12	0.0404	0.0070	**3.00E−04**	− 418.31	115.71	19567	2571
	GenADA	0.0008	0.0090	**1.17E−03**	− 142.85	44.01	663	1577
Eubacterium nodatum*	ADc12	0.4353	0.0072	**2.52E−04**	− 1240.65	338.95	95579	2571
	GenADA	0.1259	0.0025	8.82E−02	− 805.51	472.45	42817	1577
Gordonibacter*	ADc12	0.0330	0.0089	**5.57E−05**	− 254.45	63.13	16821	2571
	GenADA	0.0116	0.0039	3.30E−02	− 351.65	164.98	7135	1577
Intestinibacter*	ADc12	0.1903	0.0154	**1.01E−07**	− 3014.37	566.17	70292	2571
	GenADA	0.0024	0.0024	9.22E−02	− 246.05	146.10	2152	1577
Lachnospira*	ADc12	0.0034	0.0067	**3.30E−04**	530.79	147.83	2997	2571
	GenADA	0.0004	0.0021	1.11E−01	113.71	71.44	439	1577
LachnospiraceaeUCG008	ADc12	0.0784	0.0081	**1.08E−04**	− 776.50	200.53	35344	2571
	GenADA	0.0041	0.0017	1.57E−01	197.29	139.40	3224	1577
Oscillibacter	ADc12	0.0124	0.0068	**3.08E−04**	− 630.36	174.67	8305	2571
	GenADA	0.0051	0.0027	7.55E−02	302.62	170.24	3884	1577
Prevotella9*	ADc12	0.0084	0.0075	**1.57E−04**	− 556.34	147.21	6609	2571
	GenADA	0.0008	0.0031	5.47E−02	− 147.32	76.68	819	1577
Roseburia*	ADc12	0.2061	0.0100	**1.35E−05**	− 3564.78	819.18	78446	2571
	GenADA	0.0037	0.0027	7.64E−02	− 366.88	207.02	3135	1577
Sutterella	ADc12	0.4928	0.0071	**2.53E−04**	3594.16	982.20	124985	2571
	GenADA	0.0002	0.0009	3.08E−01	− 34.75	34.10	156	1577
**Veillonella**	ADc12	0.0070	0.0081	**8.83E−05**	539.94	137.72	5406	2571
	GenADA	0.0021	0.0085	**1.56E−03**	369.43	116.78	1843	1577

Genetic association between PRSs for top 20 microbiome genera and AD diagnosis from PRSice-2 “best-fit” model. The association from the “best-fit” threshold was generated from PRSice-2 with a range of *p*-value thresholds from 5 × 10^−8^ to 1 and incremental interval of 5 × 10^−5^; R^2^: Variance explained by the PRS model; *P*: *p*-value of model fit for the association; Coeff: Coefficient of the model; SE: standard error; # of SNPs: Number of SNPs included in the model at the specified threshold. Genera in bold are three genera identified to have a genetically significant association with AD in both discovery and replicate samples. *: indicated ten genera that have the same direction in both discovery and replicate samples. Seven genera, originally identified to be significantly associated with AD in the discovery sample, did not survive the replicate analysis due to the opposite direction in the replicate sample.

**Figure 2:**
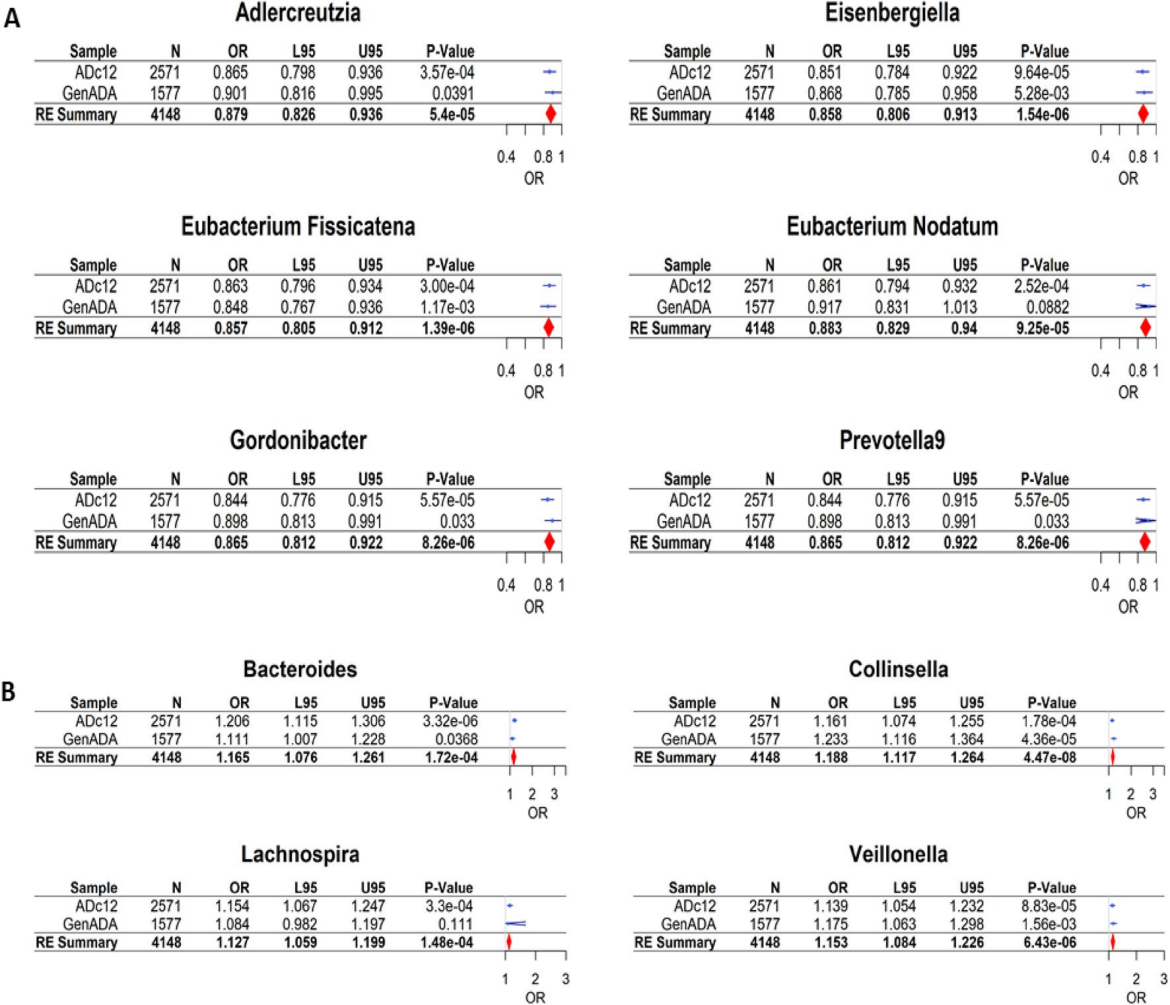
Forest plots of ten genera significantly associated with AD from meta-analysis. (**A**) The genetically predicted abundance of six genera showed significant association (*p* < 0.00042) with AD diagnosis as a protective factor with ORs < 1.0. (**B**) Conversely, four genera showed significant association with AD as a risk factor with ORs > 1.0. OR (95%CI): Odds ratio of the respective genus with the lower and upper 95% confidence intervals.

To validate our findings for the top 20 genera in the discovery sample, we further conducted the PRS analysis in the independent replication sample (GenADA). Two risk-associated genera (*Collinsella* and *Veillonella*) and one protective genus (*Eubacterium fissicatena*) remained significantly associated with AD diagnosis in the replication sample (*p* < 0.005). Ten other genera did not reach significance, but had the same effect direction as in the discovery sample (Table 3). To evaluate the overall association of the original top 20 genera from the discovery sample, we conducted a meta-analysis with the discovery and replication samples. As a result, a total of ten genera, including the three genera validated from the replication sample, were significantly associated with AD diagnosis (See Fig. 2 and Table S1).

Of the ten significant genera from the meta-analysis, six genera—*Adlercreutzia, Eubacterium nodatum group, Eisenbergiella, Eubacterium fissicatena group, Gordonibacter,* and *Prevotella9*—were identified as protective, and four genera—*Collinsella, Bacteroides, Lachnospira,* and *Veillonella*—were identified as a risk factor for AD. From the meta-analysis, *Eisenbergiella* was identified as the strongest protective factor for AD with *p* = 1.39 × 10^−6^ and OR = 0.857 (95% CI 0.805–0.912), and *Collinsella* was identified as the strongest risk factor for AD *p* = 4.47 × 10^−8^ and OR = 1.188 (95% CI 1.117–1.264).

The meta-analysis also found three genera to have a suggestive association (0.00042 < *p* < 0.05) with AD diagnosis, of which all were potential protective factors (*Intestinibacter*, *Candidatus Soleaferrea,* and *Roseburia*) (See Table S1). In addition, seven genera—*Alistipes, Anaerostipes, Catenibacterium, Eubacterium coprostanoligenes group, Lachnospiraceae UCG-008, Oscillibacter,* and *Sutterella*—originally identified to be associated with AD in the discovery sample, did not show any association in the meta-analysis due to the opposite effects in the replication sample.

Next, a multivariate logistic regression analysis, including sex, age, and two *APOE* genotypes (rs429358 and rs7412) as covariates, was used to determine any confounding effects on the association between the ten significant genera and AD diagnosis. As shown in Supplementary Table S2, the ten significant genera remained significantly associated with AD diagnosis in the discovery sample (*p* < 0.05), which suggested that the genetic association between PRSs for the ten significant genera and AD diagnosis was independent of age, sex, and *APOE* genotypes. As expected, age and *APOE* were strongly associated with AD in the multivariate logistic regression analysis. Specifically, age and rs429358 minor allele C were risk factors as shown the positive correlation with AD diagnosis, while rs7412 minor allele T was a protective factor with the negative correlation with AD diagnosis. However, sex did not show any association with AD in this study.

**Figure 3:**
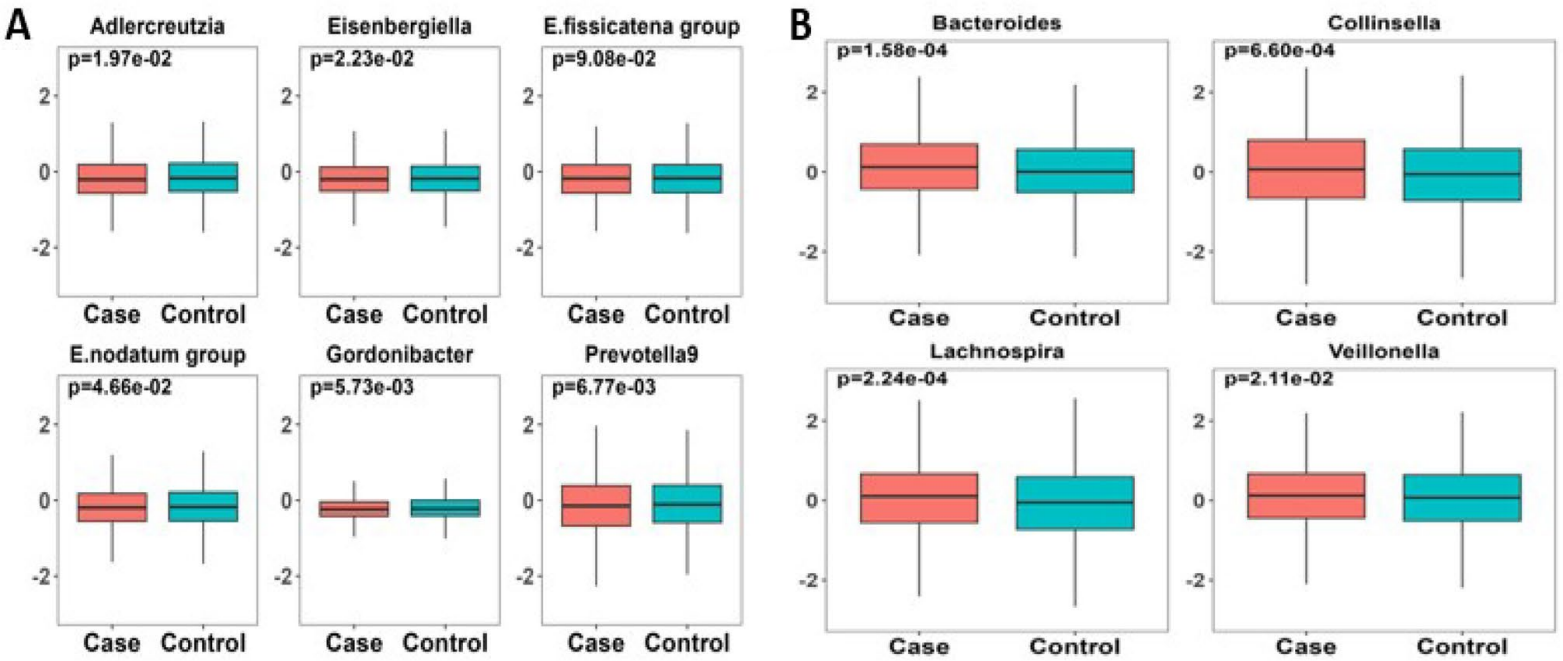
Normalized PRSs for ten significant genera between AD cases and controls in the discovery sample. (**A**) PRSs for six genera were relatively lower in AD cases than controls (*p* < 0.05), suggesting they might be a protective factor for AD. (**B)** PRSs for four genera were relatively higher in AD cases vs. controls (*p* < 0.05), suggesting they were likely be a risk factor for AD. Wilcoxon Rank Sum test was applied to generate *p* values. X-axis: Diagnosis (AD cases/controls). Y-axis: z-score normalized PRSs for each of the ten significant genera.

To better visualize the difference of PRSs from the ten significant genera between AD cases and controls, we constructed a box plot along with the Wilcoxon Rank Sum test^42^ in the discovery sample. As compared to cognitively normal controls, Fig. 3A showed that AD patients had lower PRSs for the six likely protective genera (*Adlercreutzia, Eubacterium nodatum group, Eisenbergiella, Eubacterium fissicatena group, Gordonibacter,* and *Prevotella9).* On the other hand, Fig. 3B showed AD patients had higher PRSs for the four risk genera (*Bacteroides, Collinsella, Lachnospira, and Veillonella*). These results were consistent with the PRSice-2 "best-fit" model and logistic regression analysis between PRSs and AD diagnosis.

### Correlation between PRSs for the top ten significant genera and *APOE* genotypes

*APOE* is a well-known genetic risk for AD^40,41^. Depending on the alleles of two SNPs rs429358 and rs7412, the human *APOE* gene has three alleles (ε2, ε3, and ε4)^41^. The ε4 allele is the most influential risk factor for AD beyond age; a single ε4 allele increases one’s risk by three to four folds compared with the ε2 or ε3 allele^40^. Several studies have been conducted for the potential links between the *APOE* genotypes (rs429358 and rs7412) and the gut microbiota^16–18^, but not at the genome-wide level. For this reason, we sought to determine whether there was a genetic link between the PRSs for the ten significant genera and the *APOE* genotypes. Linear regression analyses were performed between the z-score normalized best PRSs for the ten significant genera and *APOE* minor alleles at rs429358 and rs7412. The meta-analysis showed that four out of ten significant genera were correlated with *APOE* rs429358 risk allele C (*p* < 0.05) (Table 4). Notably, *Collinsella* was the only genus that was positively correlated with AD diagnosis and *APOE* risk allele C at rs429358 in both discovery and replication samples (*p* < 0.05) (Tables 3 and 4). PRSs for three genera—*Adlercreutzia, Eubacterium nodatum*, and *Prevotella9*—identified negatively correlated with AD diagnosis showed negative correlation with *APOE* risk allele C at rs429358.

**Table 4:** Association between PRSs for ten significant gut microbiota genera and *APOE* rs429358.

Trait	Samples	Coeff	SE	z-value	*P*	OR (95%CI)
**Adlercreutzia**	ADc12	− 0.051	0.031	− 1.643	0.1006	0.95 (0.89–1.01)
	GenADA	− 0.093	0.040	− 2.309	0.0211	0.91 (0.84–0.99)
	Meta-analysis	− 0.067	0.025	− 2.712	**0.0067**	0.94 (0.89–0.98)
Bacteroides	ADc12	0.019	0.031	0.608	0.5434	1.02 (0.96–1.08)
	GenADA	0.014	0.040	0.350	0.7263	1.01 (0.94–1.10)
	Meta-analysis	0.017	0.025	0.695	0.4871	1.02 (0.97–1.07)
**Collinsella**	ADc12	0.137	0.031	4.413	1.06E-5	1.15 (1.08–1.22)
	GenADA	0.118	0.040	2.930	0.0034	1.12 (1.04–1.22)
	Meta-analysis	0.130	0.025	5.283	**1.27E-7**	1.14 (1.09–1.19)
Eisenbergiella	ADc12	− 0.105	0.031	− 3.382	0.0007	0.90 (0.85–0.96)
	GenADA	0.007	0.040	0.180	0.8575	1.01 (0.93–1.09)
	Meta-analysis	− 0.052	0.056	− 0.924	0.3553	0.95 (0.85–1.06)
Eubacterium Fissicatena	ADc12	− 0.111	0.031	− 3.557	0.0004	0.90 (0.84–0.95)
	GenADA	− 0.010	0.040	− 0.252	0.8011	0.99 (0.91–1.07)
	Meta-analysis	− 0.064	0.050	− 1.270	0.2042	0.94 (0.85–1.04)
**Eubacterium Nodatum**	ADc12	− 0.097	0.031	− 3.130	0.0018	0.91 (0.85–0.96)
	GenADA	− 0.043	0.040	− 1.080	0.2804	0.96 (0.88–1.04)
	Meta-analysis	− 0.077	0.026	− 2.912	**0.0036**	0.93 (0.88–0.98)
Gordonibacter	ADc12	− 0.108	0.031	− 3.460	0.0005	0.90 (0.84–0.95)
	GenADA	− 0.014	0.040	− 0.352	0.7248	0.99 (0.91–1.07)
	Meta-analysis	− 0.064	0.047	− 1.383	0.1668	0.94 (0.86–1.03)
Lachnospira	ADc12	0.047	0.031	1.524	0.1277	1.05 (0.99–1.11)
	GenADA	0.047	0.040	1.169	0.2425	1.05 (0.97–1.13)
	Meta-analysis	0.047	0.025	1.921	0.0548	1.05 (1.00–1.10)
**Prevotella9**	ADc12	− 0.046	0.031	− 1.486	0.1373	0.95 (0.90–1.01)
	GenADA	− 0.074	0.040	− 1.836	0.0666	0.93 (0.86–1.01)
	Meta-analysis	− 0.057	0.025	− 2.299	**0.0215**	0.94 (0.90–0.99)
Veillonella	ADc12	0.092	0.031	2.946	0.0032	1.10 (1.03–1.17)
	GenADA	− 0.027	0.040	− 0.664	0.5071	0.97 (0.90–1.05)
	Meta-analysis	0.035	0.059	0.596	0.5513	1.04 (0.92–1.16)

Genera in bold are four genera identified to have genetically significant correlation with *APOE* rs429358 minor allele C in the meta-analysis (*p* < 0.05). *Collinsella* was the only genus that showed significant correlation in both discovery and replication samples.

**Figure 4:**
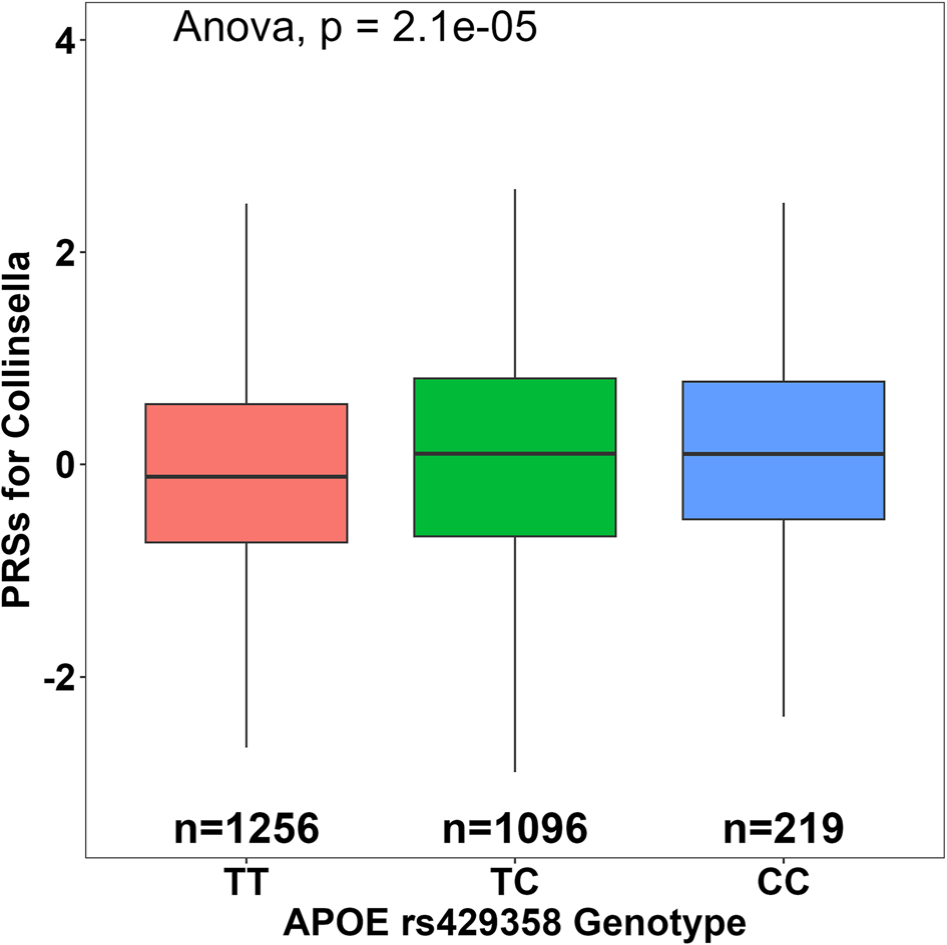
Genetic association between PRSs for *Collinsella* and *APOE* rs429358 genotype in the discovery sample. Individuals in the discovery sample were separated by their genotype at the *APOE* SNP rs429358. Those with the genotype of TC and CC had higher PRSs for genetically predicted *Collinsella* abundance than those with the TT genotype.

To illustrate the correlations between PRSs for *Collinsella* and *APOE* risk allele C at rs429358, we constructed a box plot along with ANOVA analysis. As shown in Fig. 4, a positive correlation between PRSs for *Collinsella* and *APOE* risk allele C at rs429358 was found in the discovery sample (*p* = 2.1 × 10^−5^). This positive correlation indicated that a genetic factor determining *Collinsella* abundance was more likely to occur in individuals with *APOE* minor allele C (CC and TC) as compared to individuals with two T alleles (TT) at rs429358.

Overall, our results showed that *Collinsella* was a risk factor for AD diagnosis and that* Collinsella* was positively correlated with *APOE* risk allele C at rs429358. On the other hand, three genera identified as protective factors (*Eubacterium nodatum group, Adlercreutzia,* and *Prevotella9*) for AD diagnosis showed a negative correlation with *APOE* risk allele C at rs429358 (Table 4). These associations indicate that certain microbial genera and *APOE* may contribute to disease modulation in some similar biological pathways, synergizing in disease risk or protective effects. The associations between PRSs for the four genera and the *APOE* rs429358 risk allele were independent of sex and age, as the results remained significant after adjustment for these cofactors (Supplementary Table S3). For the *APOE* genotype at rs7412, we did not see any significant correlation with the PRSs for the ten significant genera from the meta-analysis.

### Association between microbiome abundance and *APOE* genotypes

To further investigate the association between the abundance of all the gut microbiota genera and *APOE* genotypes, we retrieved summary statistics for the two *APOE* SNPs rs429358 and rs7412 directly from the 119 genera GWAS summary statistics in the MiBioGen consortium study. As shown in Table 5, rs429358 was marginally correlated with the abundance of ten genera, and rs7412 was marginally associated with the abundance of eight genera (*p* < 0.05). Together, these findings indicate that the *APOE* genotypes may have some impact on the microbiome abundance at the genus level and that the association may synergistically contribute to the risk for human diseases such as AD. Our results open the door for future studies to explore the role of the interaction between *APOE* and the gut microbiota and find a new target for treatment in human diseases.

**Table 5:** List of gut microbiome genera that were nominally associated with *APOE* SNPs rs429358 and rs7412.

rsID (CHR:BP:Effect Allele)	Microbiome Genera	Beta	SE	SZ	P	N	# cohorts
rs429358 (19:45411941:C)	Bacteroides	− 0.044	0.015	− 2.881	0.0040	18173	23
	Butyricimonas	− 0.044	0.020	− 2.241	0.0250	10657	23
	Dorea	0.030	0.015	2.127	0.0334	17494	23
	Eubacterium coprostanoligenes	0.033	0.015	2.120	0.0340	17261	23
	Faecalibacterium	− 0.035	0.015	− 2.188	0.0287	17960	23
	Olsenella	0.064	0.033	1.993	0.0462	3721	13
	Parasutterella	0.040	0.019	2.216	0.0267	11291	23
	Senegalimassilia	0.050	0.024	2.207	0.0273	6888	21
	Veillonella	0.044	0.021	2.032	0.0421	9194	23
rs7412 (19:45412079:T)	Butyricicoccus	0.048	0.021	2.468	0.0136	15637	20
	Collinsella	0.052	0.023	2.214	0.0268	12811	19
	Coprococcus3	0.050	0.022	2.344	0.0191	14323	20
	Eubacterium hallii	0.053	0.021	2.453	0.0142	14846	20
	Lachnospiraceae UCG001	− 0.071	0.027	− 2.534	0.0113	9357	20
	Olsenella	− 0.103	0.043	− 2.346	0.0190	3721	13
	Ruminococcaceae UCG004	0.080	0.028	2.901	0.0037	9049	20
	Senegalimassilia	0.082	0.032	2.584	0.0098	6508	18

SNP data were extracted from GWAS summary statistics of human gut microbiome abundance conducted from 24 multi-ethnic cohorts in the MiBioGen consortium (www.mibiogen.org)^24^. rsID (CHR:BP:Effect Allele): the rs number of single nucleotide polymorphisms, chromosome number (CHR), base pair (BP), with the effect allele (genome assembly GRCh37/hg19). Microbiome: the genus level with significant abundance associated with *APOE* two SNPs. Beta: Beta coefficient. SE: Standard Error. SZ: Weighted sum of z-scores. P: *p*-value (*p* < 0.05 was considered as nominal association between the microbiome genera and *APOE*). N: sample count. # Cohorts: Number of cohorts involved.

## Discussion

The microbiota is a complex ecosystem that comprises more than 100 trillion symbiotic microbial cells in the human body, of which 95% inhabit the human gut^44^. The bacteria from phylum Firmicutes and Bacteroidetes form a significant proportion (90%) of the adult gut microbiota, while Actinobacteria composes the rest^45^. Recently, significant evidence has shown that the gut microbiota influences normal systemic physiological homeostasis and that dysbiosis of gut microbiota may contribute to the pathogenesis of brain diseases, including AD. The gut microbiota interacts with the central nervous system (CNS) across the MGBA via microbial components, metabolic products, and neural stimulation. In this study, we leveraged extensive GWAS data to study the genetic correlation between gut microbiota genera and AD diagnosis. PRSs for 20 genera were initially found significantly associated with AD in the discovery sample, three of which were replicated in the independent replication sample. A further meta-analysis between our discovery and replication samples identified a strong genetic association between ten gut microbiota genera and AD diagnosis. Six genera were negatively associated with AD diagnosis and four genera were positively correlated with AD diagnosis. “Negative association” means that the abundance of these genera is lower in AD patients as compared to normal controls. Thus, PRSs for such genera are regarded as a protective factor for the disease. Similarly, “positive association” means that the abundance of those genera is higher in AD cases as compared to normal controls, indicating their PRSs would be seen as a risk factor against the disease. Genera identified as a protective factor were primarily from the Firmicutes phylum (*Eubacterium nodatum group, Eisenbergiella,* and *Eubacterium fissicatena group)* as well as from Actinobacteria (*Adlercreutzia*, *Gordonibacter*) and Bacteroidetes (*Prevotella9*). Positively correlated, or risk-associated genera were from phyla including Firmicutes (*Lachnospira* and *Veillonella*), Actinobacteria (*Collinsella*), and Bacteroidetes (*Bacteroides*).

In the discovery sample, the correlation of the ten significant genera remained statistically significant after being adjusted for sex, age, and two *APOE* SNPs (rs429358 and rs7412), suggesting that the genetic correlation between the ten genera and AD diagnosis was independent of age, sex, or *APOE* genotypes. In addition, we found that four of the ten significant genera showed a strong correlation with the *APOE* rs429358 risk allele C via linear regression analysis. Interestingly, the genera showing a positive correlation with *APOE* rs429358 risk allele C tend to have a positive (risk) association with AD, while the genera showing a negative correlation with *APOE* rs429358 risk allele C have a negative (protective) association with AD.

In our analyses, *Collinsella* from the phylum Actinobacteria was identified as a risk factor for AD in both the discovery and replication samples. *Collinsella* was also positively correlated with *APOE* rs429358 risk allele C in both samples. The abundance of *Collinsella* in the gut has been previously associated with rheumatoid arthritis, atherosclerosis, and Type-2 diabetes^46–48^. Importantly, an increased abundance of this genus has also been observed in AD transgenic mice and AD patients^49,50^. Our findings provide evidence at the human genome-wide level of a connection between *Collinsella* and AD that supports previous observational studies. At the molecular level, this connection is possibly driven by the pro-inflammatory effects of the *Collinsella* genus. In a human intestinal epithelial cell line, the presence of *Collinsella* increased the expression of inflammatory cytokines (IL-17A) and chemokines (CXCL1, CXCL5). *Collinsella* also increased gut permeability by reducing the expression of tight-junction proteins^51^. Furthermore, the strong association between *Collinsella* and *APOE* rs429358 risk allele C in our study may provide new insight into the pathogenesis of AD. For example, a study found that *Collinsella* correlates with higher serum levels of total cholesterol and low-density lipoprotein (LDL) cholesterol in healthy adults^52^, which may be correlated with the interaction between *Collinsella* and *APOE*. Functional studies that further explore the relationship between *Collinsella*, lipid metabolism, and inflammatory signals would help to elucidate how their interaction influences AD and other diseases.

Three genera of the Firmicutes phylum—*Eubacterium nodatum group, Eisenbergiella,* and *Eubacterium fissicatena group*—had a negative association with AD diagnosis. *Eisenbergiella, Eubacterium fissicatena group,* and *Eubacterium nodatum group* are known to contain species that metabolize the short-chain fatty acid (SCFA) butyrate from dietary carbohydrates^53–56^. Butyrate is a major SCFA metabolite in the colon that might be a critical mediator of the colonic inflammatory response. Alongside its anti-inflammatory properties, butyrate is also essential in maintaining tight junctions that prevent dysbiotic gut permeability^57,58^. Despite their production of butyrate, several studies have identified *Eisenbergiella* and *Eubacterium nodatum group* as microbial features associated with neurodegenerative diseases. A notable study of patients with AD and vascular dementia found that the gut abundance of these genera could be used to discriminate severe dementia patients against those with mild or moderate dementia^59^. High serum levels of the IgG antibody against oral *Eubacterium nodatum* were associated with lower AD risk in another study^60^. This suggests that oral and gut populations of the same microbial taxa may have different etiologies with the same disease, however, our base data covers only the gut abundance of microbiota. Nevertheless, we are the first to report a protective association between genetically-predicted *Eisenbergiella, Eubacterium nodatum group,* and *Eubacterium fissicatena group* abundance with AD, but more studies are needed to understand how these three genera may interact with the pathology of AD.

In addition, we identified two Firmicutes genera as risk factors for AD (*Lachnospira* and *Veillonella*), with *Veillonella* being validated in the replication sample. Recently, it was reported that AD patients have an abundance of *Veillonella* in their oral microbiome^61^. In the gut, it has been shown that an overabundance of species like *V.parvula* promotes intestinal inflammation by activating macrophages via the lipopolysaccharide-Toll-like receptor 4 (LPS-TLR4) pathway^62^. The dual association of oral and gut abundance of *Veillonella* with disease points to this genus as a target for therapeutics and a potential bridge between conditions like gut inflammation and periodontitis with AD. On the other hand, gut *Lachnospira* and *Veillonella* species have also been identified as beneficial or commensal to gut health, such as *Lachnospira* being protective against Crohn’s disease, or *Veillonella* interacting with *Streptococcus* species to modulate immune responses in the small intestine^63,64^. In an observational study from a Chinese group, patients with AD had decreased *Lachnospira* at the genus level compared with healthy controls^65^. However, this may reflect national differences in diet or the genetics of microbial abundance, as our study uses mostly Caucasian subjects from the United States in our discovery and replication samples.

The Bacteroidetes genera, *Prevotella9* and *Bacteroides,* were identified as protective and risk factors, respectively, in our meta-analysis. There is a complex relationship between *Prevotella* and *Bacteroides* abundance and intestinal diseases^66^. In humans, *Prevotella* is more common in populations with plant-based and high-carbohydrate diets^67^. Conversely, *Bacteroides* is more abundant in those consuming “western” diets high in protein and fat^68^. One major study showed that *Prevotella* was higher in individuals with greater adherence to Mediterranean diets, which is thought to be protective against neurodegenerative diseases^69–71^. The protective effects of *Prevotella* abundance may come from the positive dietary effects on the genus. Our association of higher genetically-predicted *Bacteroides* abundance with AD risk supports the findings of previous observational studies^11,72,73^. *Bacteroide*s species are capable of secreting LPS as an endotoxic biomolecule, which has been implicated in pathological endothelial dysfunction of the gut and can induce neuroinflammation in microglia cells^74–76^. However, it should be noted that a meta-analysis including Chinese studies found no risk association between *Bacteroides* and AD^12^, which may again reflect national differences in diet and microbial abundance.

Two protective genera, *Gordonibacter* and *Adlercreutzia,* are from the Actinobacteria phylum. These genera tend to produce metabolites beneficial to mitochondrial function, namely Urolithin A (UA) and Equol^77,78^. UA is an anti-inflammatory compound that enhances mitophagy, the removal of dysfunctional mitochondria in a cells^79^. Impaired mitophagy is part of the pathogenesis of AD, thus, UA and *Gordonibacter* species might be promising therapeutic targets against aging and AD^80^. Equol is an estrogen-like compound that reduces microglial inflammation when stimulated by LPS and downregulates genes in neurons related to apoptosis^81^. The beneficial effects of these bacterial metabolites could drive the protective association of *Gordonibacter* and *Adlercreutzia* abundance with AD that we found in this study.

The strength of our study include the use of the largest available GWASs of gut microbiota taxa to date that allow us to identify multiple genera genetically associated with AD after the strict Bonferroni correction. The use of logistic regression analysis alongside our initial PRS analyses allowed us to adjust for potential confounders, such as sex, age, and *APOE* alleles, and further validate that the association was independent of those confounders. Additionally, we are the first to study the genetic correlation between the gut microbiota and the *APOE* gene at the human genome-wide level.

## Limitations

There are several limitations to our study. First, the sample size for our base microbiome GWAS may not be large enough to truly cover the effect size of the host genetic variants, even though the MiBioGen study has a larger sample size compared to other microbiome GWASs. Because of this, we may not have enough power to detect some of the associations in our meta-analysis that were considered significant in the discovery sample. Future studies with larger sample sizes would be more capable of drawing solid conclusions about the genetic connection between gut microbiota and AD. As microbiome is highly influenced by lifestyle and environmental factors, the lack of information on these confounders in our base and target data prevents the subtyping of patients. Given the phenotypes available in our genotyping data, we included age, sex, and *APOE* genotype as covariates in our multivariate logistic regression models to account for their confounding effects. Second, our genotyping data for AD studies were mostly drawn from European American individuals, which limits the generalization of our conclusions when applied to other ethnic groups. Although the largest ethnic cohort of the MiBioGen GWAS was European (Table 1), the inclusion of other ethnic groups in the original study’s meta-analysis may be a confounding factor in our results. More diverse genotyping datasets would enable us to capture the variability in risk for AD across different ethnicities. Third, the 16S rRNA sequencing used to generate genetic associations in the “base” GWAS only provides taxa resolution from the phylum to the genus level. Fully understanding the role of bacterial taxa that may drive the pathology of AD will require methods that can capture the abundance of individual species and their mechanistic impact on the MGBA.

## Conclusions

Overall, our novel findings of ten significant genera associated with AD from the meta-analysis provide new insights into the interplay of the gut microbiota on AD. Genetic associations with the abundance of certain bacterial genera inhabiting the gut correlate with AD diagnosis in risk and protective directions. Risk-associated genera, such as *Collinsella,* have been previously tied to neuroinflammatory processes across the MGBA, while protection-associated genera like *Gordonibacter* are known to secrete metabolites that promote gut and brain health. PRSs for four genera were further identified as significant associations with the *APOE* genotype at rs429358. Our results advance the understanding of how gut dysbiosis may play a role in the pathology of AD. Future investigations with larger cohorts of AD patients from different ethnic backgrounds and more powerful microbiome GWASs are needed to better understand these genetic associations. Functional studies are also required to establish causality between particular gut microbiota and AD pathology.

## Supplementary Information

Supplementary Figure S1.

Supplementary Tables.

## Data Availability

The Full GWAS summary statistics of mbQTLs analyzed during this study are available at https://mibiogen.gcc.rug.nl/. Genotyping data for the NIA/LOAD (phs000168.v2.p2) and GenADA (phs000219.v1.p1) cohorts are available at https://www.ncbi.nlm.nih.gov/gap/. All data generated in this study are included in the manuscript, Supplementary Tables S1–3, and Supplementary Fig. S1.

## Abbreviations

AD: Alzheimer’s disease
*APOE*: Apolipoprotein E
BBB: blood–brain barrier
MGBA: microbiota-gut-brain axis
LPS: Lipopolysaccharide
TLR4: Tol-like receptor 4
SNP: single nucleotide polymorphism
GWAS: genome-wide association study
OR: odds ratio
CI: confidence interval
LD: linkage disequilibrium
PRS: polygenic risk score
CNS: central nervous system
IRB: Institutional review board
UNLV: University of Nevada Las Vegas
NIA/LOAD: National Institute of Aging/Late-onset Alzheimer’s Disease Study
GenADA: Multi-site Collaborative Study for Genotype–Phenotype Associations in Alzheimer’s Disease
NINCDS-ADRDA: National Institute of Neurological and Communicative Disorders and Stroke, and the Alzheimer's Disease and Related Disorders Association
LDL: low-density lipoprotein
SCFA: short-chain fatty acid
UA: Urolithin A
mbQTL: microbial quantitative trait loci
mbBTL: microbial binary trait loci
