# Supplementary material for: Genetic correlations between Alzheimer’s disease and gut microbiome genera
Source: Sci Rep. 2023 Mar 31;13:5258. doi: 10.1038/s41598-023-31730-5 (PMC10066300; doi:10.1038/s41598-023-31730-5)
Supplement: Supplementary file 1 — Supplementary Figure S1. [file 41598_2023_31730_MOESM1_ESM.docx]

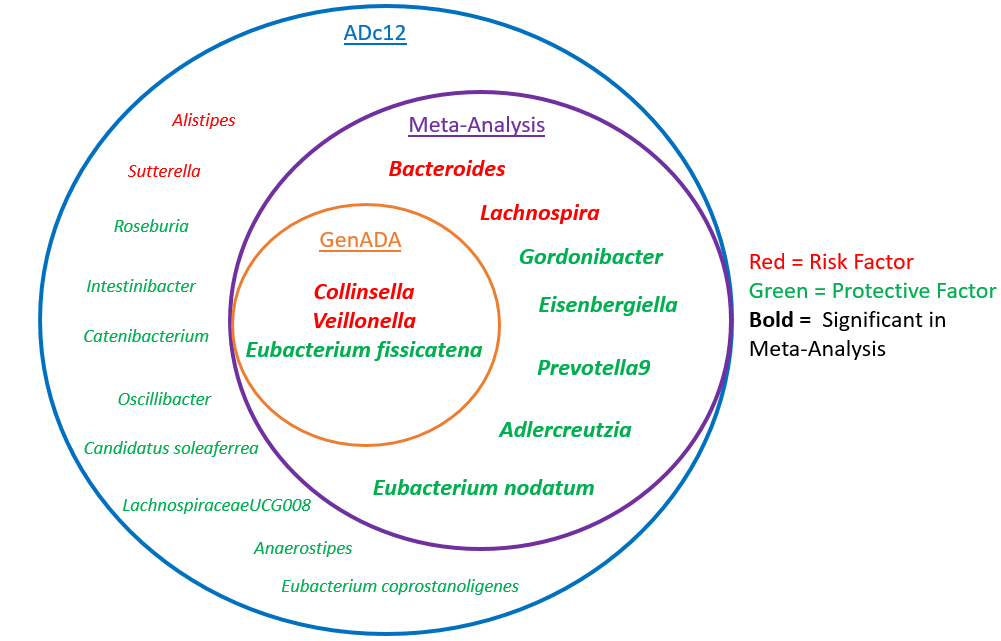


**Figure S1: Summary of significant genera across analyses**

Our initial PRS analysis in the discovery sample (ADc12-Blue) identified 20 genera significantly associated with AD in a risk direction for six genera (Red) and a protective direction for 14 genera (Green). Of these 20 genera, three remained significant in the independent replication sample (GenADA-Orange). A meta-analysis (Purple) of the 20 genera from both the discovery and replication samples identified ten genera (in bold) as significant, including the 3 from the replication sample. Of the ten genera, four were risk factors (Red) for AD and six were protective factors against AD. The ten genera outside the meta-analysis were only significant in the discovery sample.
